# Effect of Short-Term Kinesiology Taping on Knee Proprioception and Quadriceps Performance in Healthy Individuals

**DOI:** 10.3389/fphys.2020.603193

**Published:** 2020-11-11

**Authors:** Zhen Wei, Xiao-Xi Wang, Lin Wang

**Affiliations:** ^1^School of Kinesiology, Shanghai University of Sport, Shanghai, China; ^2^Tangshan People’s Hospital, Tangshan, China

**Keywords:** short-term, kinesiology taping, knee proprioception, muscle strength, muscle activation

## Abstract

**Background**: Kinesiology taping (KT) is well known measure for preventing musculoskeletal injuries. Our study aims to explore the actual effects of KT on healthy participants’ knee proprioception and quadriceps performance within 1 h.

**Methods**: A total of 35 healthy male amateur runners were recruited in our study. Four taping sequences were randomly allocated to four different weeks, namely, no taping, placebo taping, KT with tension, and KT with no tension. A CON-TREX isokinetic dynamometer was used in assessing the participants’ knee proprioception and muscle strength of knee extension and flexion at 60°/s. The electromyography (EMG) signals of medial oblique muscle and vastus lateralis were collected using Myon EMG system synchronously. Two-way repeated measures ANOVA was used in exploring the difference between taping and time effects, and the significance was set to alpha <0.05.

**Results**: Significant interaction effect was found between the taping groups and time effect [*F* (3.32) = 2.389, *p* = 0.029, *η*^2^ = 0.050] in the peak torque during the concentric contraction of quadriceps. No significant interaction and no significant differences between groups and time effects in knee proprioception and muscle activation.

**Conclusion**: The effect of KT seems insufficiently large to impose a positive effect on healthy people within short periods. Health participants may not necessarily use KT to increase muscle activation and proprioception of knee.

## Introduction

Kinesiology taping (KT), an elastic, water-resistant, and air permeable tape, was developed by Kenzo Kase in the 1970s, the tape can stretch to 120–140% of its original length and can recoil after application (Kase et al., 2003). On the basis of its special features, the compressive and shear force applied by the tape on the skin activates cutaneous mechanoreceptors related to joint position, movement, and force (Kase et al., 2003), conveying more information from the knee joint to the central nervous system for integration and, therefore, resulting in enhanced proprioception (Riemann and Lephart, 2002; Liu et al., 2019). Previous studies verified that the recoiling force direction of the tape is the same as that of a muscle contraction, is potentially transmitted to the fascia and facilitates a target muscle’s production of high force (Kuo and Huang, 2013; Yam M. L. et al., 2019; Ye et al., 2019).

Knee joint proprioception and the lower extremity muscle performance play an essential role in preventing musculoskeletal injuries (Hosp et al., 2015; Pope et al., 2017). Improvement of proprioception sensitivity and quadriceps performance may reduce the risk of injuries (Oliveira et al., 2016; Bertelsen et al., 2017; Pope et al., 2017). Given the popularity of KT among sports enthusiasts, numerous studies demonstrated that KT can improve knee proprioception (Hosp et al., 2015) and activate the quadriceps (Liu et al., 2019). However, the proposed effect of KT was not found in some studies (Fernandes de Jesus et al., 2016; Oliveira et al., 2016; Serrao et al., 2016). Poon et al. (2015) even demonstrated that KT may cause sub-optimal quadricep function (Herrington et al., 2005; Poon et al., 2015).

Most previous studies related to the KT explored the effect of KT after 0, 24, or 48 h (Lins et al., 2013; Hosp et al., 2015; Lins et al., 2016; Kim et al., 2017). Given that after taping, participants may have different physical activities within 24 h, KT may generate plastic deformations to different degrees, and the actual effect of KT may not manifest despite taping for 24 or 48 h. Additionally, common sports, such as basketball, football, and badminton, are played for 1 h, and thus taping for a day or two cannot reflect the effect of taping for sports injury prevention. The acute influence of KT on knee neuromuscular function must be investigated. According to the contradictory results of previous work comparing KT with no taping or KT without tension, multigroup studies for exploring the short-time effects of knee taping on proprioception and quadriceps performance are limited.

Seeing that running activity is common in competitive sports, running-related injuries was common in the knee joint, our study included runners and performing multiple sets of comparisons to explore difference in proprioception and quadriceps performance difference after taping at 0.5 and 1 h. We hypothesized that KT has a positive effect on knee proprioception and quadriceps performance within a short period and can act as a novel technology for preventing musculoskeletal injuries.

## Materials and Methods

### Participants

A total of 35 healthy males were recruited for this study (aged, 22.5 ± 2.6 years; body height, 176.4 ± 5.3 cm; and body mass, 71.2 ± 7.8 kg). The sample size was estimated by G∗Power (G∗Power 3.1.9.2, Heinrich-Heine-Universität Düsseldorf, Düsseldorf, Germany^1^) based on *α* level of 0.05, and power (1-*β*) of 0.80, and 0.15 dropout rate. The participants were all healthy people with exercise habits (running 10–15 km per week) from the local university. All of them were right-leg dominant according to their preferred leg when kicking a ball (Ghena et al., 1991). Participants with lower limb musculoskeletal injuries or other medical conditions 6 months before the study were excluded. The participants provided informed consent before the experiments. This study was approved by the Human Ethics Committee of Shanghai University of Sport (2018073).

### Taping Methods

After the height and body mass of each participant were measured, each participant sat with the hip and knee joint of the dominant limb in 90° flexion. As suggested by Kase et al. (2003), the taping direction was from the proximal to the distal. Four taping sequences were randomized in a counterbalanced order: (1) no taping (NT), (2) placebo taping (PT), (3) KT with tension (KT-T), and (4) KT with no tension (KT-NT). To prevent any carryover effect (Fu et al., 2008), the total experiment for each participant lasted for 4 consecutive weeks, 1 week of washout phase was performed between each taping condition.

The taping methods of the KT-T on rectus femoris (RF), vastus medialis oblique (VMO), and vastus lateralis (VL) were as follows (Lins et al., 2013; Poon et al., 2015; Fernandes de Jesus et al., 2016): (1) from a point 10 cm below the anterior superior iliac spine and split in the form of “Y” above the patella, finishing at tibial tuberosity (RF); (2) from the middle third of the medial thigh to the medial edge of the patella (VM); and (3) start from the greater trochanter of femur, finishing at the lateral edge of the patella (VMO). KT-T consistency with 50% tension was maintained in the muscles with the following equation, the leg length of the taping area was measured to calculate the actual length of the taping (Yam M. L. et al., 2019; Yam T. T. T. et al., 2019).

$$\mathrm{Actual length of tape to}\;\mathrm{cut}\;\left(\mathrm{cm}\right)=\left[\left(\frac{x-4}{1.5}\right)+4\right]\times 1.1$$

where *𝑥* is the actual length of the leg, the anchor length of the KT was set to 4 cm (2 cm for the proximal and distal sites).

Taping regions of KT-NT was the same with KT-T but without any tension. For the PT condition, a 10 cm KT without tension was used 10 cm below the anterior superior iliac spine and 10 cm upper edge of the patella (Thelen et al., 2008; Fernandes de Jesus et al., 2016).

### Measurement of the Knee Proprioception and Quadriceps Performance

A CON-TREX isokinetic dynamometer (PHYSIOMED CON-TREX TP1000, Germany) was used in our study, which was reliable for assessing proprioception and isokinetic knee strength (Konig et al., 2004). The participants took the proprioception test first and then the muscle strength test after taping for 0, 0.5, and 1 h repeatedly. The intraclass correlation coefficients between the isokinetic muscle strength test were 0.96 reported by Vithoulka et al. (2010).

Proprioception was measured through joint position sense reproduction, and the participants were seated in the dynamometer chair, with their legs hanging freely. The rotation axis of the dominant leg was aligned with the lateral epicondyle of the femur. The moving arm was adjusted and fixed 5 cm above the medial malleolus according to previous studies (Dvir, 2004). Before the test, the dynamometer was calibrated to absolute zero, and the gravity correction factor was eliminated. Then each participant wore a blindfold and earplugs to eliminate auditory or visual inputs. The dominant leg of each participant was passively extended to a target angle (30°) from the starting position of 90° flexion. During this process, they were asked to relax completely to prevent active muscle contraction. The participants memorized the target angle for 5 s (Hosp et al., 2015; three times) and then moved their leg back to the starting position passively. After a pause of 5 s, the participants were requested to find the target angle positively (Cho et al., 2015). This process was repeated three consecutive times. The study parameter was the average absolute error of the three repetitions compared with the target angle.

For the assessment of the knee extensor/flexor torque, participants’ back was slightly reclined and the dominant leg was stabilized at the distal femur, the pelvic and thorax regions were also secured with a belt (Vico et al., 2013). Before the test, participants would familiarized themselves with the equipment and then performed four maximum concentric and eccentric knee joint contractions at 60°/s. Concentric contraction means that the knee joint started with the knee flexed at 90° and then extend to knee flexion at 30° positively, eccentric contraction from the knee flexion at 30–90° negatively (Ng and Wong, 2009; Padulo et al., 2013; Poon et al., 2015). Both contractions included one familiarization test using submaximal contractions. The torque parameters were recorded by means of the isokinetic dynamometer. Quadricep fatigue was minimized by giveing the participants 3 min to rest between series (Oliveira et al., 2016). Moreover, verbal encouragement was given during the test (Fernandes de Jesus et al., 2016). The time to peak torque, peak torque and average power were recorded for contractions for further data analysis, and the peak torque was normalized for body weight and expressed in percentage (PT/BW * 100).

The electromyography (EMG) signals of VMO and VL were captured synchronously using a Myon 320 wireless EMG system with 16-channel conditioning module (Myon 320, Schwarzenberg, Switzerland) during the muscle strength test, which was accurate to collect EMG activities (Yin et al., 2020). The skin was shaved and cleansed with 70% alcohol. Then, we attached a self-adhesive surface electrode with a diameter of 2 cm on the longitudinal axis of the muscle belly according to the European recommendations of Hermens et al. (2000). The sampling frequency was configured at 2000 Hz, and the signal was filtered between 20 and 500 Hz and then preprocessed using a full-wave rectifier. The root mean square (RMS) approach was used at a window size of 100 ms. The EMG device was connected to a laptop, which received the signal and stored in a file. Pro-EMG software was used for the digital analysis of the signals. For the normalization of RMS values, the participants were tasked to perform two maximum voluntary isometric contractions (MVIC) when their knees were at 60° flexion for 5 s; they were asked to rest every 1 min (Burden, 2010).

### Statistical Analysis

All data are presented as mean and standard deviation (SD). The Kolmogorov-Smirnov test was used to test the normal distribution. Two-way repeated ANOVA was used in exploring the differences in interaction and main effects between the taping groups over time. Statistical significance was set at alpha <0.05. Bonferroni *post hoc* tests were conducted to compare specific differences. All statistics were performed using SPSS 22 (IBM, Armonk, NY, United States).

## Results

**Figure 1:**
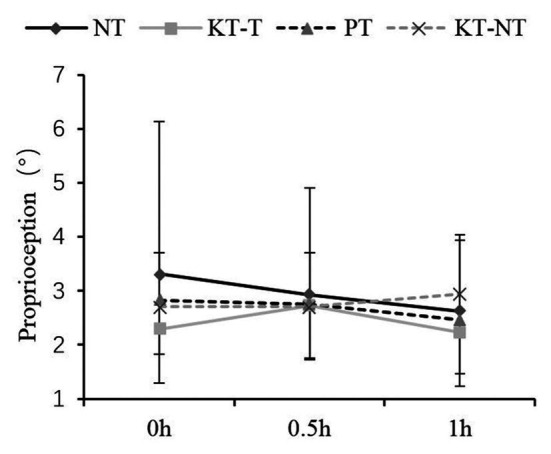
Comparison of the knee proprioception after taping within short times.

With regard to knee joint proprioception, no significant interaction effect was found between the taping and short-time effect groups (Figure 1). *Post hoc* analysis revealed that the four taping methods produced similar absolute errors within 0, 0.5, and 1 h (*p* = 0.288).

We only found a significant interaction effect between the taping and time effect groups [*F* (3.32) = 2.389, *p* = 0.029, *η*^2^ = 0.050] in peak torque during the concentric contraction of the quadriceps (Table 1), but *Post hoc* analysis showed no difference among the taping methods within short periods. No significant interaction effect was found for the time to peak torque (*p* = 0.363) and average power (*p* = 0.091) during the concentric contraction of the quadriceps.

**Table 1:** Comparison of the muscle strength after taping within short times.

			NT	KT-T	PT	KT-NT	*F*	*P*	*η*^2^
Concentric	TPT (s)	0 h	0.49 ± 0.15	0.49 ± 0.13	0.48 ± 0.11	0.47 ± 0.13	1.018	0.363	0.007
		0.5 h	0.52 ± 0.15	0.48 ± 0.12	0.47 ± 0.13	0.48 ± 0.12	1.113	0.355	0.024
		1 h	0.48 ± 0.12	0.47 ± 0.15	0.48 ± 0.14	0.47 ± 0.13	0.183	0.908	0.004
	PT (N*m)	0 h	2.09 ± 0.54	2.21 ± 0.54	2.16 ± 0.51	2.17 ± 0.49	2.389	0.029^*^	0.050
		0.5 h	2.07 ± 0.49	2.21 ± 0.55	2.14 ± 0.52	2.17 ± 0.45	0.180	0.910	0.004
		1 h	2.25 ± 0.50	2.19 ± 0.51	2.11 ± 0.51	2.22 ± 0.41	1.863	0.157	0.014
	AP (w)	0 h	74.07 ± 22.66	79.37 ± 26.76	79.33 ± 27.48	80.14 ± 26.98	1.840	0.091	0.039
		0.5 h	74.13 ± 23.17	81.37 ± 24.98	81.10 ± 29.76	79.19 ± 28.19	0.167	0.919	0.004
		1 h	82.35 ± 26.96	80.26 ± 22.74	80.76 ± 28.44	80.76 ± 24.64	2.806	0.062	0.020
Eccentric	TPT (s)	0 h	0.48 ± 0.12	0.49 ± 0.12	0.50 ± 0.09	0.48 ± 0.13	0.623	0.711	0.014
		0.5 h	0.48 ± 0.11	0.50 ± 0.12	0.49 ± 0.12	0.50 ± 0.12	0.094	0.963	0.002
		1 h	0.50 ± 0.11	0.50 ± 0.13	0.51 ± 0.12	0.48 ± 0.13	0.685	0.505	0.005
	PT (N*m)	0 h	2.43 ± 0.60	2.44 ± 0.65	2.42 ± 0.58	2.40 ± 0.66	0.641	0.697	0.014
		0.5 h	2.45 ± 0.61	2.51 ± 0.59	2.50 ± 0.62	2.43 ± 0.68	0.040	0.989	0.001
		1 h	2.50 ± 0.62	2.44 ± 0.57	2.41 ± 0.50	2.44 ± 0.56	1.359	0.259	0.010
	AP (w)	0 h	91.41 ± 22.87	92.80 ± 25.55	93.39 ± 21.36	90.51 ± 27.64	0.849	0.533	0.018
		0.5 h	93.85 ± 24.06	95.08 ± 19.87	92.05 ± 20.35	91.89 ± 26.25	0.073	0.974	0.002
		1 h	2.50 ± 0.62	2.44 ± 0.57	2.41 ± 0.50	2.44 ± 0.56	0.857	0.425	0.006

Values are means ± standard deviation (SD); significant differences (^*^p < 0.05) are highlighted in bold for interaction effect, taping effect, and time effect from the top for different parameters. TPT, time to peak torque; PT, peak torque; and AP, average power.

Under the eccentric contraction of the quadriceps, the time to peak torque (*p* = 0.711), peak torque (*p* = 0.697), and average power (*p* = 0.533) parameters were similar between taping and time effects (Table 1). *Post hoc* analysis showed no significant difference among the four taping methods within 0.5 and 1 h.

**Figure 2:**
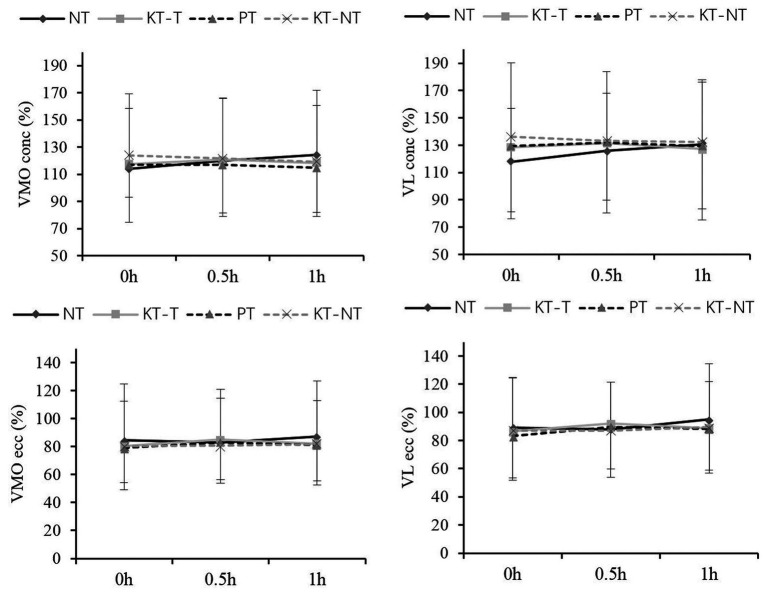
Comparison of the electromyography (EMG) activities of vastus medialis oblique (VMO) and vastus lateralis (VL) after taping within short times. Means and standard deviations of the variables: normalized root mean square (RMS) of the VMO and VL during concentric (conc) and eccentric (ecc) contraction.

None of the VMO and VL muscle activities during the concentric and eccentric contraction analyzed showed significant intergroup or intragroup differences (Figure 2).

## Discussion

The present study provides an intuitive way of investigating the actual effect of KT on knee joints within short periods. The results reveal that the knee proprioception and muscle activation cannot be improved by the application of KT. Data from our research may guide health individuals in using KT to prevent knee injuries during exercises.

Shortly after taping, no significant difference on knee proprioception was found among the four taping groups, indicating that KT cannot improve knee proprioception in health people. This result is supported by the results of previous studies that explored knee joint position sense in healthy subject after taping for 24 h (Torres et al., 2016). However, Liu et al. (2019) established that KT can improve the knee proprioception of patients with anterior cruciate ligament (ACL) injury. Cho et al. (2015) found that KT potentially improves positive knee joint position sense in patients with knee osteoarthritis after 1 h. Callaghan et al. (2008) showed that the poor proprioception of patients with patellofemoral pain syndrome can be improved after taping. For people with knee ACL injury (Barrack et al., 1994), knee osteoarthritic (Barrett, 1991) or knee with chronic effusions (Wingfield, 2013; Achenbach et al., 2017), injury history may be contributed to knee position sense receptor deficiency even after recovery. Poor proprioception may be more sensitive to and easier to facilitate with KT and transfer more proprioceptive information from the joint structures to the nervous system. Therefore, patients with poor proprioception tend to receive more benefits than healthy participants with good proprioception (Callaghan et al., 2002; Hosp et al., 2015). As a result, healthy knee joints may not require KT to prevent knee injuries during competitions, but its use by people with knee injuries history is reasonable. This hypothesis requires further investigation because of insufficient high-quality research exploring the effect of KT under different sports durations.

Muscle strength did not change within 1 h, indicating that the pulling force that was transmitted to the fascia and facilitated the target muscle cannot impose any obvious effect on healthy people. Furthermore, the elasticity of the KT may gradually decrease and even persist for a long time but cannot impose any effect on knee joint. The results are partly identical with those of Melo et al. (2018) and Lins et al. (2016), who showed that KT cannot alter muscle strength during the knee extension process after taping for 0 or 72 h compared with NT. Given that the studies in the current literature mainly explored the effect of KT within 24 or 48 h, the short time effects of KT should be studied specifically.

A significant interaction effect on peak torque during the concentric contraction was found, but muscle strength was the same in the four taping groups within short periods, indicating that the recoil force of KT cannot increase muscle strength. This result was partly supported by a recent study of Yeung and Yeung (2016), who explored the excitability of the quadriceps’ motor neurons by comparing the latency time of the knee-jerk reflex and proposed that KT can produce force for the quadriceps, although they reported that the effect is not enough to alter muscle contraction strength. Additionally, the positive interaction effect may be related to psychological comfort (Donec and Kubilius, 2020). Poon et al. (2015) included a placebo group to explore the psychological comfort of KT and demonstrated that KT cannot improve the muscle strength and EMG activity of quadriceps during an isokinetic muscle strength test and even suspected that the positive results reported by previous studies may be attributed to psychological effects. Although the PT group in our studies was similar to the KT group, we still speculated that psychological factors play an important role in taping, but the underlying physiological mechanisms were not fully clarified.

In the current study, no significant change in the eccentric contraction of quadriceps was observed. Although Vithoulka et al. (2010) demonstrated that the pulling force generated by KT can facilitate the fascia and plays an important role in the mechanical transmission of muscle eccentric contraction. Our results and those of Oliveira et al. (2016) suggested that the potential effect generated by KT cannot produce a positive effect within short periods or even within 24 h. The discrepancy may be partially explained by psychological comfort, as shown by the results of Yeung and Yeung (2016).

With respect to EMG activity, even though the theories indicated that KT can activate skin receptors and improve neuromuscular recruitment, our study revealed no significant taping or time differences. VMO and VL activity did not change after taping for 0.5 or 1 h. The finding partly supports the results of previous studies where no significant difference was observed after taping 24 or 48 h (Lins et al., 2016). This study also supported other recent studies indicating that the stimulation of KT cannot activate quadricep activity (Lins et al., 2013; Oliveira et al., 2016; Serrao et al., 2016; Melo et al., 2018). However, our findings contradicted the data presented by Watanabe et al. (Herrington et al., 2005; Watanabe, 2019), who proposed that the surface EMG amplitude of the VL muscle decreased and the application of a tape may decrease the neuromuscular activation of the knee extensor muscles in healthy runners. Moreover, the inhibited effect of patellar taping was also verified in patients with patellofemoral pain syndrome by Ng and Wong (2009), who found that VMO and VL activities in patients with knee injuries significantly decrease after patella taping and concluded that knee taping may cause the joint to function sub-optimally. Therefore, the current study and previous data suggest that taping cannot produce a positive effect on knee joint through the activation of muscle activity, which may be inhibited as in the case of patellar taping.

A limitation of the present study was that the results can only represent the effect of KT on healthy peoples’ knee proprioception and muscle performance. The actual effect of KT on sports enthusiasts with knee injury history requires further study.

## Conclusion

In the current study, subtle stimulation produced by KT may not cause a measurable change in function in healthy participants within short periods. Therefore, compared with individuals with knee history, healthy sports enthusiasts may cautiously use KT to improve their neuromuscular function.

## Data Availability Statement

The original contributions presented in the study are included in the article/supplementary material, and further inquiries can be directed to the corresponding author.

## Ethics Statement

The studies involving human participants were reviewed and approved by Human Ethics Committee of Shanghai University of Sport (2018073). The patients/participants provided their written informed consent to participate in this study.

## Author Contributions

ZW recruited the subjects, collected the data, and wrote the manuscript. X-XW and LW conceived the study, undertook statistical analysis, and interpreted the results. All authors contributed to the article and approved the submitted version.

### Conflict of Interest

The authors declare that the research was conducted in the absence of any commercial or financial relationships that could be construed as a potential conflict of interest.

## Footnotes

^1^http://www.gpower.hhu.de/
